# A multi-institutional real world data study from India of 3453 non-metastatic breast cancer patients undergoing upfront surgery

**DOI:** 10.1038/s41598-020-62618-3

**Published:** 2020-04-03

**Authors:** Dinesh Chandra Doval, Selvi Radhakrishna, Rupal Tripathi, Renu Iyer Kashinath, Vineet Talwar, Ullas Batra, Naga Amulya Mullapudi, Kapil Kumar, Ajay Kumar Dewan, Harit Chaturvedi, Juhi Tayal, Anurag Mehta, Sudeep Gupta, Ramesh B. V. Nimmagadda

**Affiliations:** 1 Department of Medical Oncology, Rajiv Gandhi Cancer Institute & Research Centre, Delhi, India; 2 Department of Surgical Oncology, Chennai Breast Centre, Chennai, Tamil Nadu India; 3 Department of Research, Rajiv Gandhi Cancer Institute & Research Centre, Delhi, India; 4 Department of Medical Oncology, Apollo Cancer Institutes, Chennai, Tamil Nadu India; 5 Department of Surgical Oncology, Fortis Hospital, Delhi, India; 6 Department of Surgical Oncology, Rajiv Gandhi Cancer Institute & Research Centre, Delhi, India; 7 Department of Surgical Oncology, Max Hospital, Delhi, India; 8 Department of Research (Biorepository), Rajiv Gandhi Cancer Institute & Research Centre, Delhi, India; 9 Department of Laboratory and Transfusion Services, Rajiv Gandhi Cancer Institute & Research Centre, Delhi, India; 10 Department of Medical Oncology, Tata Memorial Centre, Homi Bhabha National Institute, Mumbai, India

**Keywords:** Breast cancer, Cancer epidemiology

## Abstract

The present analysis reports the clinical, pathological, treatment profile and overall survival (OS) and disease-free survival (DFS) outcomes of consecutive breast cancer patients from three Indian centres, who underwent curative surgery as their first treatment. Among the 3453 patients, stage I, II, and III cases were 11.75%, 66.79%, and 21.64%, respectively while hormone receptor positive/HER2 negative, triple negative (TNBC) and hormone receptor any/HER2 positive cases were 55.2%, 24.2% and 20.6%, respectively. The five-year OS in the entire cohort, node-negative and node-positive patients were 94.1% (93.25–94.98), 96.17% (95.2–97.15) and 91.83% (90.36–93.31), respectively, and the corresponding DFS were 88.1% (86.96–89.31), 92.0% (90.64–93.39) and 83.93% (82.03–85.89), respectively. The five-year OS in hormone receptor positive/HER2 negative, TNBC and HER2 subgroups were 96.11% (95.12–97.1), 92.74% (90.73–94.8) and 90.62% (88.17–93.15), respectively, and the corresponding DFS were 91.59% (90.19–93.02), 85.46% (82.79–88.22) and 81.29% (78.11–84.61), respectively. This is the largest dataset of early breast cancer patients from India with survival outcome analysis and can therefore serve as a benchmark for future studies.

## Introduction

Breast cancer, an increasing public health dilemma, represents a heterogeneous group of diseases and still remains today the leading cancer in women and a major cause of morbidity and mortality worldwide^1^. As per the GLOBOCAN 2018 statistics, there were a total of 1157294 new cancer cases in the year 2018 in both sexes across all ages in India. Among these, there were 162468 cases of breast cancer with a mortality of 87090 cases. The five-year prevalence across all age groups was 405456 cases^2^. A high-incidence cancer of high-resource nations, breast cancer has swiftly become a global healthcare challenge.

Improvements in screening, imaging, awareness and diagnostic strategies have led to improvement in the early detection of the disease. Owing to the advent of newer regimes and options, treatment stratification has greatly developed in the past decade^1^. A multitude of therapeutic options have been developed and tested leading to major oncologic breakthroughs. Molecular based classifications and personalized treatment have also evolved in the past decade. This has also led to improvements in overall, progression-free and relapse-free survival^3,4^. Nevertheless, a patient’s survival is related to several prognostic factors, including number of positive lymph nodes, tumor size, hormone receptor status, histological type and grade, and patient’s age^5^. Although much has been reported by the Western world, it is imperative to have a large population-based data to study the disease characteristics of Indian patients with breast cancer with their unique profile in order to have more tailored treatment strategies with the ultimate goal of improving the survival and quality of life of these patients.

The present study assessed the clinical, pathological, treatment and survival profile of breast cancer patients who had undergone surgery as the first modality of definitive treatment.

## Materials and Methods

A multi centric retrospective study was conducted and data was collected from patients with breast cancer who were enrolled in one of the three participating institutions namely Rajiv Gandhi Cancer Institute & Research Centre (RGCI&RC), Delhi; Apollo Cancer Institutes (ACI), Chennai and Chennai Breast Centre (CBC), Chennai, India. During the study period 2008 to 2014, a total of 4918 patients were registered with a diagnosis of breast cancer, of which 4409 patients eventually had treatment and follow up at one of the participating centers. Of these, after excluding carcinoma *in situ* and sarcoma cases, 3453 (78%) patients undergoing upfront surgery were included in the study for detailed analysis. RGCI&RC, ACI and CBC contributed 2296, 215 and 942 patient’s anonymized data, respectively in the final analysis. The study was approved by the Institutional Review Board/ Ethics Committee of RGCI&RC and CBC (vide letters dated 10.09.2013 and 23.03.2019, respectively) and granted waiver to ACI (vide letter dated 20.09.2018). The study was conducted as per the Helsinki Declaration. Prior to starting treatment, the patients gave a written, informed consent for using their data for research/ publication. None of the researchers named in the author list of the paper had access to identifying patient information when analysing the data.

Medical records were referred to for culling out the data and extracting patient information. Data was collected and collated related to demographic profile, tumor details, pathologic assessments, treatment and follow up information. Status at last follow up was confirmed either through medical records or telephonically.

Breast cancer staging was done as per the TNM AJCC 7^th^ Edition guidelines^5^. For the purpose of pathologic analysis, immune histochemical staining was done on paraffin sections and the expression levels of estrogen receptor (ER), progesterone receptor (PR) and human epidermal growth factor receptor 2 (HER2) were assessed. The test sample was scored using the ASCO-CAP guidelines (2007) of ER/PgR and HER2 with reference to the internal control. HER2 2+ cases were confirmed by Fluorescent *In-Situ* Hybridization (FISH) for amplification.

Data was collected in OncoCollect data collection software and Microsoft R Open software version 3.5.1 was used for statistical analysis. Survival analysis was performed using the Kaplan Meier method^6^. Log rank test was applied for comparing the survival differences between the groups. A two-sided p-value < 0.05 was considered as significant.

## Results

A total of 3453 patients with breast cancer were included in the study. The median age at diagnosis was 53 years (20–89 years). The clinical and tumor profile of these patients is shown in Table 1. Among these, 98.6% patients were females and 60% of the patients were postmenopausal. The number of patients with left and right sided tumor was comparable (51% & 49%, respectively). The median pathological tumor size was 3 cm (0–16 cm). Infiltrating ductal carcinoma (IDC) or invasive breast cancer (IBC) NOS histology (94%), pathological stage IIA (40.9%), tumor grade 2 (49.8%), lymph node negativity (52.4%), absence of lymphatic invasion (67.7%) and no extra capsular spread in node positive tumors (52.4%) was most commonly observed. The incidence of infiltrating lobular carcinoma was ~2% in our patient group. The median positive lymph node ratio was 0.15 (0.02–1). The majority of the patients were ER positive (64.1%), PR positive (57.8%) and HER2 negative (74.6%) and hence the most common receptor subgroup was hormone receptor positive/HER2 negative (1751/ 3174, 55.2%).

**Table 1:** Clinical and tumor profile of 3453 patients with early breast cancer.

Characteristics	N	n (%)
*Median age in years (Range)*	3453	53 (20–89)
*Sex*	3453	
Female		3403 (98.6)
Male		50 (1.4)
*Menstrual status*	3394	
Postmenopausal		2038 (60)
Premenopausal		1356 (40)
*Primary side*	3429	
Left		1749 (51)
Right		1680 (49)
*Histology*	3453	
IDC or IBC NOS		3246 (94)
ILC		79 (2.3)
Medullary		11 (0.3)
Mucinous		61 (1.8)
Others		56 (1.6)
*pT Size in cm*	3269	
<2 cm		670 (20.5)
2.1–3 cm		1323 (40.5)
3.1–5 cm		1037 (31.7)
>5.1 cm		239 (7.3)
*Pathological stage*	3379	
I		397 (11.8)
IIA		1381 (40.9)
IIB		876 (25.9)
IIIA		427 (12.6)
IIIC		298 (8.8)
*Grade*	3293	
1		299 (9.1)
2		1640 (49.8)
3		1354 (41.1)
*Lymphatic invasion*	3227	
No		2186 (67.7)
Yes		1041 (32.3)
*Positive nodes*	3413	
Zero		1788 (52.4)
1–3		949 (27.8)
4–9		398 (11.7)
>10		278 (8.1)
*Positive node ratio*	1625	
Median		0.15 (0.02–1.0)
*Extra capsular spread in node positive*	1474	
Negative		772 (52.4)
Positive		702 (47.6)
*ER*	3421	
Negative		1228 (35.9)
Positive		2193 (64.1)
*PR*	3422	
Negative		1444 (42.2)
Positive		1978 (57.8)
*HER2 IHC 2* +	554	
Negative		309 (55.8)
Positive		105 (19)
Unclassifiable		140 (25.2)
*Receptor subgroups*	3174	
ER/PR + HER2−		1751 (55.2)
ER/PR +/− HER2+		654 (20.6)
Triple negative		769 (24.2)

IDC, invasive ductal carcinoma; IBC NOS, invasive breast cancer not otherwise specified; ILC, invasive lobular carcinoma; pT, pathological tumor size; ER, estrogen receptor; PR, progesterone receptor; HER2, human epidermal growth factor receptor 2; IHC, immunohistochemistry; N, number.

Table 2 shows the treatment profile of the patients included in the study. Majority of the patients underwent mastectomy (78.7%) while axillary dissection had been performed in 87.1% patients. In terms of chemotherapy, anthracycline alone or in combination with taxane drugs had been administered in 83.6% patients who received adjuvant chemotherapy. Tamoxifen used alone or sequentially with aromatase inhibitor endocrine therapy in hormone receptor positive patients was most commonly given (51.3%). A total of 41.8% patients had been irradiated and the therapy was given most commonly in both the primary site and the nodes (29.9%) as compared to the primary site alone (11.9%).

**Table 2:** Treatment profile of 3453 patients with early breast cancer.

Characteristics	N	n (%)
*Surgery*	3418	
Breast conserving surgery		727 (21.3)
Mastectomy		2691 (78.7)
*Axillary surgery*	3422	
No surgery		107 (3.1)
Axillary dissection		2978 (87.1)
Sentinel node		337 (9.8)
*Adjuvant chemotherapy drugs*	3453	
No chemotherapy		810 (23.5)
Anthracycline based		701 (20.3)
Anthracycline-taxane based		1510 (43.7)
Taxane based		304 (8.8)
Others		128 (3.7)
*Chemotherapy cycles*	2292	
< 4		165 (7.2)
5–6		1887 (82.3)
>7		240 (10.5)
*Endocrine therapy in hormone receptor positive*	1673	
Aromatase inhibitors		815 (48.7)
Tamoxifen		738 (44.1)
Tamoxifen switched toAromatase inhibitors		120 (7.2)
*Adjuvant Radiotherapy*	3453	
No		2010 (58.2)
Yes		1443 (41.8)
*Adjuvant Radiotherapy site*	3453	
No Radiotherapy		2010 (58.2)
Primary site		411 (11.9)
Primary site and nodes		1032 (29.9)
*Adjuvant Radiotherapy in node positive*	1645	
No		675 (41)
Yes		970 (59)
*Adjuvant Trastuzumab*	562	
No		302 (53.7)
Yes		260 (46.3)

N, number.

Profile of patients with early breast cancer on the basis of menopausal status has been shown in Table 3. Hormone receptor positivity was more commonly observed in postmenopausal patients (59%) whereas triple negativity was more prominent in premenopausal group (31%). Positive lymph nodes and tumor grade 3 were commonly seen in the premenopausal patients (50% and 46%, respectively). Comparisons between menopausal status and receptor subgroups (p-value < 0.001), lymph nodes (p-value 0.03), tumor grade (p-value < 0.001) and pathological stage (p-value 0.004) were observed to be statistically significant.

**Table 3:** Profile of patients with early breast cancer on the basis of menstrual status.

Characteristics	N	Postmenopausal n (%)	Premenopausal n (%)	p-value
*Primary side*	3373			0.243
Left		1047 (52)	671 (50)	
Right		976 (48)	679 (50)	
*Breast p stage*	3324			**0**.**004**
I		263 (13)	128 (10)	
IIA		829 (41)	525 (40)	
IIB		514 (26)	349 (26)	
IIIA		235 (12)	187 (14)	
IIIC		162 (8)	132 (10)	
*Tumour grade*	3242			**<0**.**001**
1		216 (11)	79 (6)	
2		992 (51)	616 (48)	
3		746 (38)	593 (46)	
*Lymphatic invasion*	3175			0.351
No		1310 (68)	842 (67)	
Yes		605 (32)	418 (33)	
*Lymph nodes*	3377			**0**.**03**
Negative		1085 (54)	672 (50)	
Positive		941 (46)	679 (50)	
*Receptor subgroups*	3129			**<0**.**001**
ER/PR + HER2−		1106 (59)	610 (48)	
ER/PR + /− HER2+		386 (21)	264 (21)	
Triple negative		375 (20)	388 (31)	

ER, estrogen receptor; PR, progesterone receptor; HER2, human epidermal growth factor receptor 2; N, number.

A higher grade 3 tumor was most frequently observed in triple negative cancer (65%) whereas grade 1 and 2 tumors were associated with hormone receptor (HR) positive tumors (73%). Lymph node positivity and higher pathological stage (stage 3) was least common in triple negative tumors (38% and 14%, respectively). Statistical comparisons between receptor subgroups and menopausal status, tumor grade, lymphatic invasion, lymph nodes, adjuvant chemotherapy drugs and pathological stage were highly significant (p-values < 0.001).

**Figure 1:**
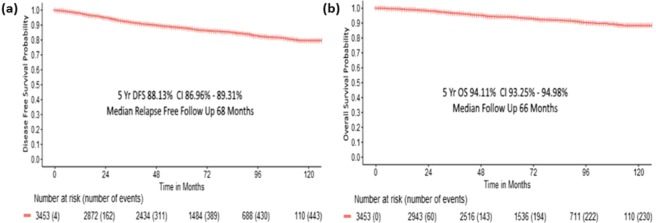
Survival patterns of the whole cohort (**a**) Disease free survival (**b**) Overall survival.

**Figure 2:**
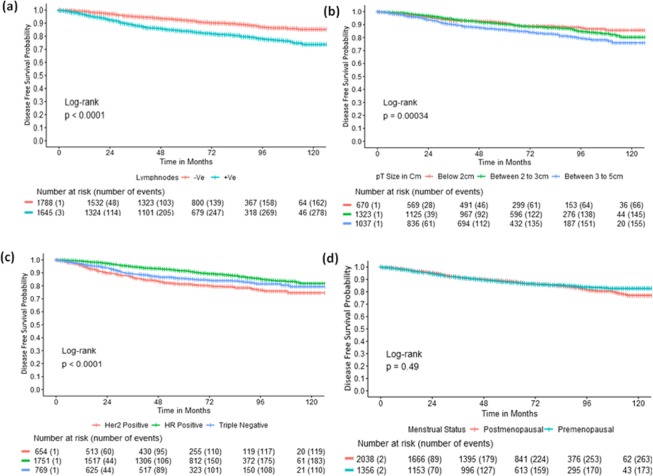
Disease free survival with respect to (**a**) lymph node status (**b**) tumor size (**c**) hormone receptor status (**d**) menstrual status.

Survival analysis of the patients is shown in Figs. 1 and 2 and Table 4. The 5 year overall survival (OS), calculated from the date of diagnosis to death, of the patients was 94.1% at a median follow up of 66 months [interquartile range (IQR) 46–91]. The disease free survival (DFS), calculated from the date of diagnosis to the date of recurrence of disease or death from any cause, whichever occurred first, was 88.1% at a median follow up of 68 months (IQR 48–92). Overall, around 19% patients were lost to follow up at 60 months for DFS, of which, 6% (31.5%) and 8.5% (44.5%) were lost to follow up at 6 and 12 months. Also, 213/328 (64.9%) relapsed patients were lost to follow up in the first month after relapse.

**Table 4:** Five Year Disease Free and Overall Survival.

Characteristics	N	5 year DFS (95% CI)	5 year OS (95% CI)
*Whole Cohort*	3453	88.1% (86.96–89.31)	94.1% (93.25–94.98)
***pT size in cm***			
<2 cm	670	91.1% (88.77–93.45)	96.9% (95.47–98.38)
2.1–3 cm	1323	90.3% (88.61–92.1)	95.5% (94.22–96.7)
3.1–5 cm	1037	85.9% (83.61–88.25)	92.4% (90.63–94.2)
>5.1 cm	239	80.9% (75.74–86.45)	87.4% (82.89–92.2)
***Nodal subgroups***			
Node Negative	1788	92% (90.64–93.39)	96.2% (95.2–97.15)
Node Positive	1645	83.9% (82.03–85.89)	91.8% (90.36–93.31)
***Receptor subgroups***			
ER/PR + / HER2−	1751	91.6% (90.19–93.02)	96.1% (95.12–97.1)
ER/PR Any/ HER2 +	654	81.3% (78.11–84.61)	90.6% (88.17–93.15)
Triple Negative	769	85.5% (82.79–88.22)	92.7% (90.73–94.8)
***Receptor/Nodal subgroups***			
ER/PR +/ HER2−/ Node +	861	88.7% (86.39–91.01)	94.4% (92.72–96.11)
ER/PR +/ HER2−/ Node −	882	94.4% (92.8–96.1)	97.7% (96.63–98.75)
ER/PR Any/ HER2 +/Node +	358	75.9% (71.22–80.81)	87.3% (83.45–91.28)
ER/PR Any/ HER2 +/ Node −	295	87.9% (83.95–92.11)	94.4% (91.65–97.32)
Triple Negative/ Node +	294	79.5% (74.61–84.66)	90.1% (86.42–93.98)
Triple Negative/ Node −	472	89.6% (86.62–92.64)	94.6% (92.31–96.87)
***pT Size/Nodal subgroups***			
<2 cm/Node +	251	86.8% (82.48–91.34)	96.2% (93.64–98.83)
<2 cm/Node −	415	93.9% (91.38–96.49)	97.7% (96.05–99.29)
2.1–3 cm/Node +	596	87.5% (84.65–90.5)	94.3% (92.21–96.35)
2.1–3 cm/Node −	726	92.5% (90.48–94.66)	96.4% (94.88–97.88)
3.1–5 cm/Node +	568	81.6% (78.15–85.13)	89.6% (86.79–92.42)
3.1–5 cm/Node −	465	91% (88.26–93.95)	95.7% (93.76–97.74)
>5.1 cm/Node +	142	75.1% (67.81–83.14)	83.4% (76.75–90.58)
>5.1 cm/Node −	97	89.4% (83–96.21)	93% (87.73–98.59)
***pT1N0/Receptor status***			
ER/PR +/HER2−	238	97.5% (95.3–99.7)	99.1% (97.79–100)
ER/PR Any/HER2+	68	88.9% (80.72–97.82)	100% (100–100)
Triple Negative	71	86.5% (78.27–95.68)	92.8% (86.25–99.89)

pT, pathological tumor size; ER, estrogen receptor; PR, progesterone receptor; HER2, human epidermal growth factor receptor 2; N, number; DFS, disease free survival; OS, overall survival; CI, confidence interval.

The 5-year OS of node-negative and positive patients were 96.2% (95.2–97.15) and 91.8% (90.36–93.31), respectively (p-value < 0.0001) whereas the DFS were 92% (90.64–93.39) and 83.9% (82.03–85.89), respectively (p-value < 0.0001). The OS and DFS were also calculated in the different receptor subgroups as per the receptor types. The hormone receptor-positive group had the highest 5 year OS (96.1%, p-value 0.0013) and DFS (91.6%, p-value < 0.0001) as compared to the HER2 positive and triple-negative groups. When the hormone receptor-positive group was sub-categorized according to their lymph node status, the 5-year OS and DFS was better in the node negative patients [97.7% (96.63–98.75) & 94.4% (92.8–96.1); p-value 0.04 & <0.0001, respectively]. A similar trend was observed in the HER2 positive (p-values 0.017 & 0.00038, respectively) and triple-negative patients (p-values 0.033 & 0.00016, respectively) also. Among the pT1N0 patients, OS and DFS were better in the hormone receptor positive group (98.5%, p-value 0.14 and 96.8%, p-value 0.0051, respectively).

The 5 year DFS and OS were also calculated with respect to the different factors as shown in Table 4. The 5 year DFS for the whole cohort was 88.1% whereas the OS was 94.1%. Further, improved 5 year DFS and OS were observed in pathological tumor size <2 cm (91.1% and 96.9%), node negativity (92% and 96.2%), ER/PR positivity HER2 negativity (91.6% and 96.1%),

The Cox proportional hazard analysis (univariate) for DFS has been shown in Table 5. The hazard ratios were statistically significant with reference to various factors including breast surgery (p-value 0.034), pathological tumor size (p-value < 0.001), tumor grade (p-value < 0.003), lymphatic invasion (p-value < 0.001), lymph nodes (p-value < 0.001), receptor subgroups (p-value < 0.035) and anthracycline containing adjuvant chemotherapy drugs (p-value 0.025).

**Table 5:** Cox Proportional Hazard Analysis (Univariate) for Disease free survival.

Characteristics	Hazard Ratio	Lower CI	Upper CI	p-value
***Breast surgery***				
Mastectomy	1	—	—	—
BCS	0.762	0.593	0.98	0.034
***pT size in cm***				
> 5.1 cm	1	—	—	—
<2 cm	0.476	0.326	0.695	<0.001
2.1-3 cm	0.537	0.384	0.75	<0.001
3.1-5 cm	0.778	0.558	1.085	0.139
***Tumour grade***				
3	1	—	—	—
1	0.265	0.154	0.456	<0.001
2	0.739	0.608	0.897	0.002
***Lymphatic invasion***				
Yes	1	—	—	—
No	0.651	0.534	0.794	<0.001
***Lymph nodes***				
Positive	1	—	—	—
Negative	0.501	0.413	0.608	<0.001
***Receptor subgroups***				
ER/PR Any/ HER2 +	1	—	—	—
ER/PR +/ HER2−	0.515	0.409	0.649	<0.001
Triple Negative	0.756	0.583	0.98	0.034
***Adjuvant chemotherapy drugs***				
Others	1	—	—	—
Anthracycline based	0.582	0.362	0.934	0.025
Anthracycline-taxane based	0.758	0.488	1.176	0.216
Taxane based	0.727	0.428	1.236	0.239
No chemotherapy	0.924	0.583	1.466	0.737
***Endocrine therapy in hormone receptor positive***				
Aromatase inhibitors	1	—	—	—
Tamoxifen	0.954	0.701	1.298	0.765
Tamoxifen switched to Aromatase inhibitors	0.538	0.261	1.109	0.093

BCS, Breast conserving surgery; pT, pathological tumor size; ER, estrogen receptor; PR, progesterone receptor; HER2, human epidermal growth factor receptor 2; CI, Confidence interval.

Further, Cox proportional hazard analysis (multivariate) was performed for DFS (Table 6). Variables like pathological tumor size [HR 1.010 (1.003–1.018), p-value 0.004], Tumor Grade [HR 1.454 (1.222–1.729), p-value < 0.001] lymph node status [HR 0.531 (0.420–0.671), p-value < 0.001] and adjuvant chemotherapy drugs [HR 1.135 (1.040–1.239), p-value 0.004] were significant.

**Table 6:** Cox Proportional Hazard Analysis (Multivariate) for Disease free survival.

Characteristic	Hazard Ratio	Lower CI	Upper CI	p-value
Breast surgery	1.078	0.938	1.239	0.292
pT size in cm (continuous)	1.010	1.003	1.018	0.004
Tumor grade	1.454	1.222	1.729	<0.001
Lymphatic invasion	1.011	0.805	1.270	0.926
Lymph nodes	0.531	0.420	0.671	<0.001
Receptor subgroups	0.992	0.905	1.087	0.859
Adjuvant chemotherapy drugs	1.135	1.040	1.239	0.004

pT, pathological tumor size; CI, Confidence interval.

## Discussion

To our knowledge, this is the largest cohort of non-metastatic breast cancer patients from India with survival outcomes. Of note, this cohort only includes patients who have undergone surgery as their first treatment and does not include and hence is not representative of locally advanced cases that usually undergo neoadjuvant chemotherapy prior to surgery. In a previously published data from India, a retrospective audit evaluated the 5 year survival and associated prognostic factors in breast cancer patients, of which, 622 patients underwent upfront surgery^7^. The outcomes reported in the present analysis specifically in patients with early breast cancer including those in clinically and biologically relevant subgroups establish a benchmark against which future studies from India and similar countries can be planned.

Mastectomy was done in 78% patients. Multiple factors are responsible for the lower rates of breast conservation surgery. Majority of patients pay out of pocket for medical treatment with only about 20% of patients covered by insurance. Quite often, the coverage was not adequate to cover all the modalities of treatment. Radiotherapy facilities are limited to metropolitan cities and tier II cities. Cost of travel, stay and loss of work for accompanying bread winners increases the cost of treatment and therefore mastectomy is seen as a cost effective option. The possible re-excision after lumpectomy for involved or close margins is also a deterring factor. The potential need for a re-excision with its financial implications makes women choose mastectomy. Besides, mastectomy is perceived to be safer by patients, their families and referring Physicians. Breast reconstruction rates after mastectomy is also lower in our practice (less than 2%). Implants for reconstruction are not popular in India. Added cost of reconstruction, possible delay in initiating systemic therapy, need for radiotherapy are all deterring factors for primary reconstruction.

Sentinel lymph node biopsy for axillary staging has been widely adopted in most parts of the world. Only 9% patients had sentinel lymph node biopsy in our study. This study included patients treated between 2008 to 2014 and in this period, a cautious approach to axillary staging was practiced in India. Since then, most centres including ours have adopted sentinel lymph node biopsy for clinically node-negative early breast cancer patients.

Patients in this study were from the middle/ high income group in society, with higher disease awareness, as well as understanding of the importance of timely and appropriate treatment and follow up. This is reflected in the higher compliance with treatment, improved follow up and better survival. The 5 year OS was 94.11% (95% CI 93.25–94.98) and DFS was 88.13% (95% CI 86.96–89.31). The survival patterns overall did not highlight any differences arising from varying basic treatment strategies. In a study by Raina *et al*., 487 early breast cancer patients were analyzed and the 5 year DFS and OS were 73% and 78%, respectively^8^. Follow up in our country is a challenge due to socioeconomic factors that include (but not restricted to) distances, stigma of cancer, dependency on family members for access to and continuity of care. In our cohort, despite being from a middle/ high income group in society, 19% patients were lost to follow up at 60 months for DFS. Patients not seen in the last 1 year were considered lost to follow up. Interestingly, many patients were lost to follow up immediately after completing the treatment; among the patients lost to follow up, 31.5% and 44.5% were lost to follow up at 6 and 12 months. Emphasizing the need for follow up and implementing suitable methods to contact patients after completing treatment will help in the long-term follow-up. Rajaraman Swaminathan *et al*. also reported the highest proportion of lost to follow-up patients in the first year from Madras Metropolitan Tumor Registry at Cancer Institute (Women’s India Association) between 1990–1999^9^. Moshim Kukar *et al*. reported 18% lost to follow-up at 5 years from a cohort of breast cancer patients who underwent surgery at Roswell Park Cancer Institute between 1997–2010^10^. These reports show that lost to follow-up is not uncommon in clinical practice outside of clinical trials. This is further compounded in our country with a relapse in the cancer. Many are lost to follow-up after a relapse, as they may choose to get treated locally or at different institutions. Some patients may also try alternative treatments. This is clearly seen in our study with 64.9% of 328 relapsed patients being lost to follow-up within the first month of documented relapse. This is reflected in the median follow-up of surviving patients being 66 months while non-relapsing patients was 68 months. Given this fact, in our setting, DFS is a more reliable endpoint in assessing treatment related outcomes. It is therefore imperative that DFS data is reported for any cohort treated with a curative intent in India. This may indeed be true in most of the low and middle-income countries.

Dinshaw *et al*. reported a study from India involving 1022 patients and identified a set of factors for loco regional recurrence including age <40 years, axillary node metastasis, lympho-vascular invasion, adjuvant systemic therapy, inner quadrant tumor and axillary node metastasis^11^. Further, Nair *et al*. also reported that the factors adversely affecting DFS in EBC were node metastasis, increasing number of metastatic nodes, hormone receptor negative and Her2neu positive status^7^. In agreement with the existing literature, our analysis confirms the prognostic significance of tumor size and nodal status. Although receptor-defined subgroups were significantly prognostic in our univariate analysis, this was lost after adjustment for covariates in the multivariate analysis. The exact reasons for this finding are unclear but it is possible that the larger average tumor size and higher rate of nodal positivity in our cohort possibly overwhelmed the prognostic impact of receptors. The prognostic impact of treatment variables (type of surgery, chemotherapy regimen, etc.) cannot be reliably evaluated from our analysis. The choice of treatment was likely influenced by the tumor and patient characteristics and therefore the analysis by treatment is likely confounded. For example, patients with larger tumors are (more) likely to have undergone mastectomy in preference to breast conservation.

The incidence of infiltrating lobular carcinoma has been found to be low in the Indian population (~2% in our patient group). This is similar to the incidence reported by the other cited literature from India [~2.4% as reported by Nigam *et al*.^12^, ~2.5% as reported by Gogia *et al*.^13^]. This may be due to the fact that population-based screening does not exist in India and it is possible that the fraction of patients with lobular carcinoma is different in screen-detected populations.

A high percentage of patients in our cohort had tumors larger than 3.1 cm (39%) and node-positive disease (47.6%). This reflects the late presentation to healthcare system in the natural history of disease and lack of public awareness. Tumor size was known to be a strong independent predictor of prognosis in invasive breast cancer, irrespective of the biological subgroups^14–16^. In a prospective nationwide population based study in Netherlands, Saadatmand *et al*. showed that tumor size and nodal status still affect the overall mortality, independent of age and tumor biology, especially with advancements in conservative surgeries and systemic therapies^17^. In our study, the outcome of patients was significantly better in patients with tumor size less than 3 cm than in patients with tumor size more than 3.1 cm. Rosen *et al*. in their study of T1N0M0 and T2N0M0 patients reported no difference in recurrence-free survival for tumor size 1.1–2.0 cm and 2.1–3.0 cm at 10 and 20 years^18^. This is particularly relevant in our country. Fancellu *et al*. in their study emphasized on the use of campaigns aimed at increasing adhesion to mammography screening and concluded that breast cancer patients in these programs had a higher probability of receiving less invasive surgery with shorter hospital stay^19^. However, while the rest of western world is still debating population-based mammographic screening for breast cancer^20–23^, Switzerland has moved away from population-based mammographic screening^24^. It would be interesting and instructive to evaluate the effect of early detection strategies such as clinical breast examination in this population as is being done in 2 large randomized trials in India^25,26^. It is also noteworthy that in line with the previous reports^27^, our cohort has higher fraction (24%) of patients with the triple-negative cases as compared to that reported from the developed countries. The exact reasons are unclear but could include younger average age of patients, lack of population-based screening, larger tumor size and others like yet-unidentified environmental, lifestyle or genetic predisposition factors. Sihto *et al*. compared the molecular subtype frequencies in population-based mammography screening and outside of screening and observed more luminal A subtype, less HER2 + /ER-, smaller tumor size and lower histological grade in the mammography screened cancers^28^.

The strength of the analysis is inclusion of patients from multiple centers in India and the large sample size which enables relatively precise estimates of survival even in subgroups. It should be noted that all the participating centers are private/ trust institutions which cater to relatively affluent sections of the Indian society. Therefore, a probable limitation of this study could be that the results may not be entirely representative of cohorts with higher fraction of underprivileged patients as seen in public sector institutions.

In summary, the report establishes the survival outcomes in the largest cohort of patients with early breast cancer from India who underwent surgical resection as their first treatment. Overall, the present study brings to light the fact that in patients undergoing upfront surgery survival is good and follow ups can be maintained. With a large percentage of the population still having tumors more than 3 cm and the DFS being good for the tumors below 3 cm and the incidence of breast cancer being relatively less as compared to the western population, it is envisaged that population-based mammographic screening is not advisable in India. It is more important to make the women and physicians aware of the importance of breast physical examination and for both to shed the inhibition of the same-which is a major factor in the country. Special emphasis is required to establish procedures to ensure timely follow-up of the patients. In India and other low and middle-income countries, it, therefore, becomes imperative to collect real world data and look at the patient characteristics, treatment and outcomes in depth, also taking into account their socio-economic status and demographic details.
